# Δ122p53, a mouse model of Δ133p53*α*, enhances the tumor-suppressor activities of an attenuated p53 mutant

**DOI:** 10.1038/cddis.2015.149

**Published:** 2015-06-11

**Authors:** T L Slatter, N Hung, S Bowie, H Campbell, C Rubio, D Speidel, M Wilson, M Baird, J A Royds, A W Braithwaite

**Affiliations:** 1 Department of Pathology, Dunedin School of Medicine, University of Otago, Dunedin, New Zealand; 2 Children's Medical Research Institute, University of Sydney, Westmead, New South Wales, Australia; 3 Department of Microbiology and Immunology, School of Medical Sciences, University of Otago, Dunedin, New Zealand; 4 Maurice Wilkins Centre for BioDiscovery, University of Otago, Dunedin, New Zealand

## Abstract

Growing evidence suggests the Δ133p53*α* isoform may function as an oncogene. It is overexpressed in many tumors, stimulates pathways involved in tumor progression, and inhibits some activities of wild-type p53, including transactivation and apoptosis. We hypothesized that Δ133p53*α* would have an even more profound effect on p53 variants with weaker tumor-suppressor capability. We tested this using a mouse model heterozygous for a Δ133p53*α*-like isoform (Δ122p53) and a p53 mutant with weak tumor-suppressor function (mΔpro). The Δ122p53/mΔpro mice showed a unique survival curve with a wide range of survival times (92–495 days) which was much greater than mΔpro/- mice (range 120–250 days) and mice heterozygous for the Δ122p53 and p53 null alleles (Δ122p53/-, range 78–150 days), suggesting Δ122p53 increased the tumor-suppressor activity of mΔpro. Moreover, some of the mice that survived longest only developed benign tumors. *In vitro* analyses to investigate why some Δ122p53/mΔpro mice were protected from aggressive tumors revealed that Δ122p53 stabilized mΔpro and prolonged the response to DNA damage. Similar effects of Δ122p53 and Δ133p53*α* were observed on wild-type of full-length p53, but these did not result in improved biological responses. The data suggest that Δ122p53 (and Δ133p53*α*) could offer some protection against tumors by enhancing the p53 response to stress.

The p53 tumor suppressor is most important for preventing cancers. p53 controls cell fate in response to stress by inducing apoptosis, cell cycle arrest/senescence, DNA repair (reviewed in Braithwaite *et al.,*^1, 2^ Oren,^3^ and Speidel^4^) or possibly restricting supply of basic substrates for metabolism.^5, 6, 7^ The regulation of p53 function has recently become more complex with the discovery of 13 isoforms, which may interfere with the normal functioning of full-length (FL) p53.^8, 9, 10, 11, 12, 13, 14^ An alternative promoter in intron 4 generates the Δ133p53 isoforms (Δ133p53*α*, and with additional alternative splicing in intron 9, Δ133p53*β*, and Δ133p53*γ*^11^).

The Δ133p53*α* isoform is expressed in many tissues, but elevated levels have been found in several cancers.^11, 15, 16^ Although the function(s) of Δ133p53*α* are not fully understood, growing evidence suggests it may have tumor-promoting capacities. Reducing Δ133p53*α* levels in the U87MG glioblastoma cell line reduced its ability to migrate and stimulate angiogenesis.^17^ Δ133p53*α* may also interfere with the tumor-suppressor functions of FLp53. The zebrafish ortholog of Δ133p53*α*, Δ113p53, inhibited p53-mediated apoptosis,^18^ and overexpression of Δ133p53*α* inhibited p53-directed G_1_ cell cycle arrest.^16^

Previously, we reported the construction and characterization of a mouse expressing an N-terminal truncation mutant of p53 (designated Δ122p53) that is very similar to Δ133p53*α*, providing the first mouse model of the Δ133p53*α* isoform.^19, 20^ Δ122p53 was found to increase cell proliferation and in p53 null cells transduced with a Δ122p53 expressing retrovirus, inhibited the transactivation of *CDKN1a* (encoding) p21^CIP1^ and *MDM2* by FLp53.^19, 20^ As well as elevating cell proliferation, homozygote Δ122p53 mice exhibited a profound pro-inflammatory phenotype, including increased serum interleukin-6 (IL-6) and *γ*-interferon (*γ*-IFN), and features of autoimmune disease.^19, 20^ The mice were tumor-prone displaying a complex tumor spectrum, but predominantly B-cell lymphomas and osteosarcomas. Thus, most evidence supports a role for the Δ133p53*α* isoform as a dominant oncogene that may interfere with normal FLp53 tumor-suppressor functions, but also has additional 'gain-of-function' properties to promote tumor progression, probably through inflammatory mechanisms.^21^

Given the above data, we reasoned that in an environment where p53 tumor-suppression capacity is compromised, such as in the context of the R72P allele^22, 23, 24^ or where p53 levels are reduced,^25, 26, 27^ the influence of Δ133p53*α* isoform on FLp53 function would be greater, leading to rapid tumor formation with a phenotype that would resemble that of the isoform alone. To test this, we generated mice heterozygous for Δ122p53 and a p53 mutant (mΔpro) that we previously described, that has attenuated tumor-suppressor activity.^28, 29^ The mΔpro mouse model is missing part of the p53 proline rich domain (PRD, amino acids 58–88). These mice are defective for DNA damage-induced apoptosis, and show a delayed and impaired cell cycle arrest response. Homozygous mΔpro mice develop late onset follicular B-cell tumors, while mΔpro heterozygotes developed few tumors in the presence of a wild-type p53 allele, or an early onset T-cell lymphoma in a p53-null background. In the latter case, the onset and tumor spectrum are indistinguishable from p53-null mice.^28^

In the current study, we found that, in contrast to our hypothesis, many Δ122p53/mΔpro mice showed extended survival compared with Δ122p53 homozygotes. *In vitro* analyses to explain this phenomenon suggested that Δ122p53 allele can enhance mΔpro tumor-suppressor functions, in particular cell cycle arrest.

## Results

### mΔpro inhibits proliferation and pro-inflammatory cytokines induced by Δ122p53

**Figure 1:**
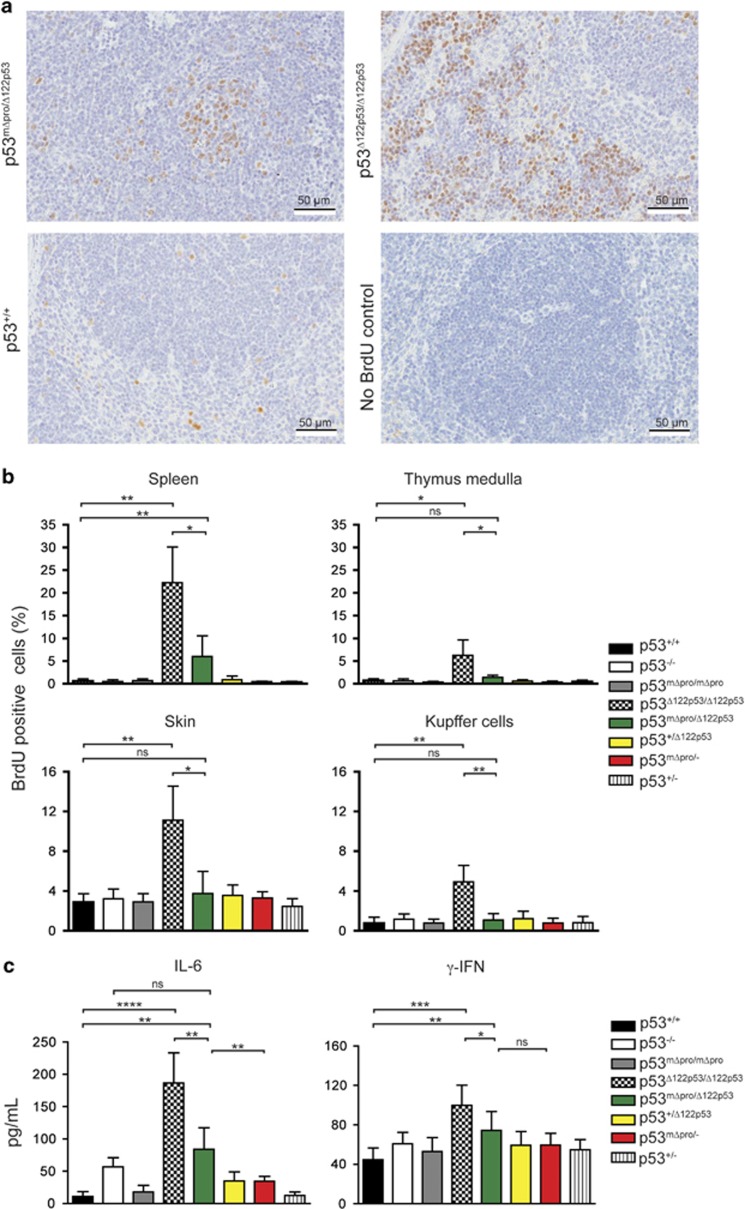
mΔpro overrides the pro-proliferative pro-inflammatory features of Δ122p53. (**a**) Examples of BrdU staining on spleen tissue from p53^+/+^, Δ122p53/mΔpro mice, and Δ122p53 mice. Mice were pulse-labeled with BrdU for 90 min to label proliferating cells. Organs were harvested and BrdU-positive cells were detected with a horseradish peroxidase-labeled antibody and light microscopy. (**b**) Quantitation of BrdU-positive cells in different tissues in Δ122p53/mΔpro mice to illustrate a reduction in the percentage of proliferating cells compared with Δ122p53 homozygote mice. Mice of various p53 genotypes were pulse-labeled with BrdU and tissues collected at necropsy. BrdU-positive cells were identified using immunohistochemistry and light microscopy and the percentage of BrdU-positive cells over the total cell count calculated. Results are represented as the mean±S.D.; *n*=4 mice per genotype. (**c**) Quantitation of serum IL-6 and *γ*-IFN by ELISA in Δ122p53/mΔpro mice to illustrate a reduction in the pro-inflammatory phenotype compared with Δ122p53 homozygote mice. In all analyses, other genotypes with Δ122p53, mΔpro, wild-type (+) or p53-null (-) alleles were included for comparison. Results are represented as the mean±S.D.; *n*, at least 4 mice per genotype. *P<0.05; **P<0.01; ***P<0.001; ****P<0.0001

Enhanced proliferation in multiple tissues and elevated levels of pro-inflammatory cytokines are profound features of homozygous Δ122p53 mice,^19^ which very likely contribute to the tumor phenotype. To determine whether mΔpro affects these activities of Δ122p53, we carried out *in vivo* proliferation assays and measured serum cytokine levels in Δ122p53/mΔpro and other mice. Examples of the BrdU staining for Δ122p53/mΔpro spleen is shown in Figure 1a and the quantitation of BrdU positive cells is shown in Figure 1b. Results showed that Δ122p53/mΔpro mice had a higher frequency of proliferating cells in the spleen compared with all genotypes (*P*<0.01) with the exception of Δ122p53/Δ122p53, but had a similar frequency in all other tissues.

Δ122p53/mΔpro mice had increased IL-6 compared with p53^+/+^, mΔpro/mΔpro, mΔpro/-, Δ122p53/+, and p53^+/−^ mice (*P*<0.01); decreased IL-6 compared with Δ122p53/Δ122p53 (*P*=0.0015), and no significant difference to p53^−/−^ mice (Figure 1c). For the analysis of *γ*-IFN, Δ122p53/mΔpro mice had increased *γ*-IFN levels compared with p53^+/+^ mice (*P*=0.0093), but they were lower compared with homozygous Δ122p53 mice (*P*=0.0137), and not significantly different to all other genotypes (Figure 1c).

Collectively, the data show that although mΔpro is weakly tumor-suppressive,^19^ it still appears to largely override the proliferative and inflammatory capacity of Δ122p53.

### Δ122p53/mΔpro heterozygous mice have an extended lifespan

**Figure 2:**
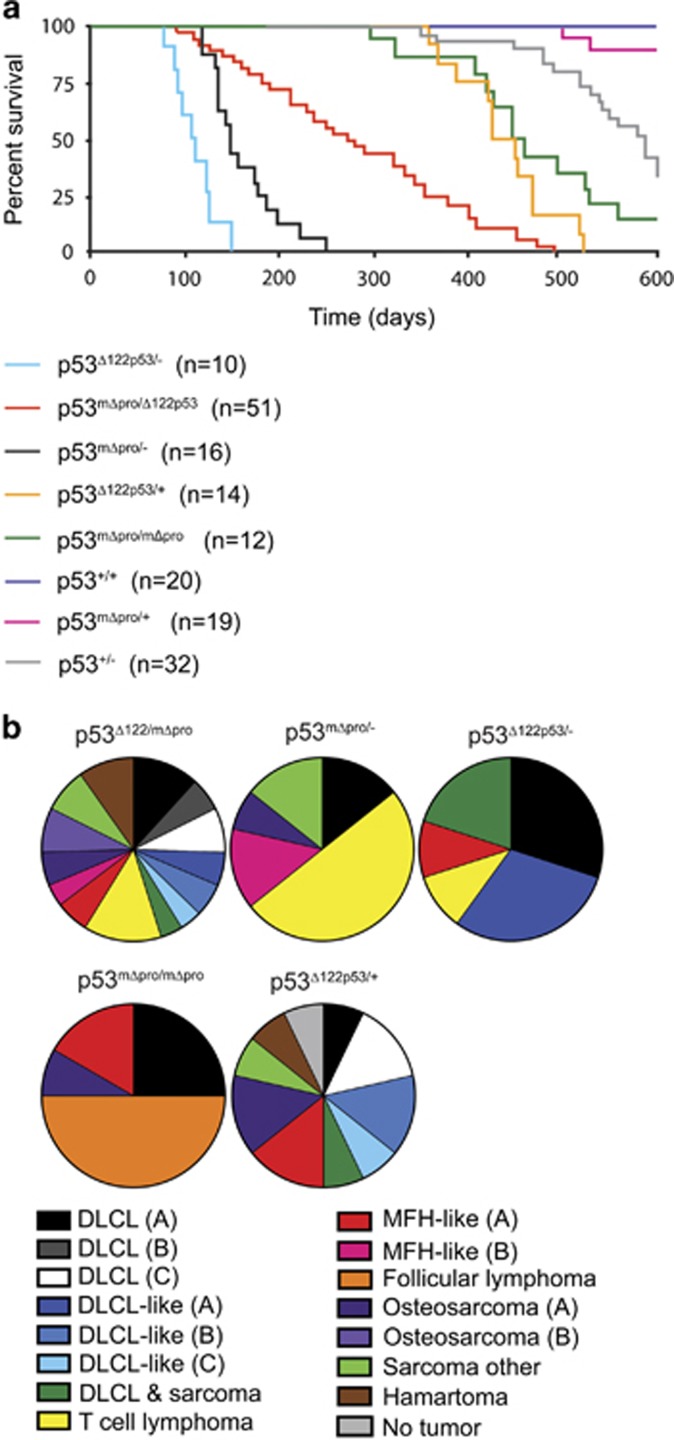
Broad lifespan and mixed spontaneous tumor spectrum of Δ122p53/mΔpro mice. (**a**) Kaplan–Meier survival curve of Δ122p53/mΔpro mice and mice with various genotype combinations (Δ122p53, mΔpro, wild-type (+), or p53-null (-) alleles and heterozygous combinations). Mice were monitored for 600 days. *n*=cohort size. (**b**) The tumor spectrum of Δ122p53/mΔpro mice in comparison with the other genotypes as identified by histo- and immuno-pathological examination. DLCL and DLCL-like tumors were further sub-grouped by cell surface markers into the following: DLCL-A, (B-cell-positive for CD34, CD10, CD45, CD45R, and CD20, and negative for CD138); DLCL-B (B-cell-positive for CD20, CD45, and CD45R, and negative for CD34, CD10, and CD138); DLCL-C (B-cell-positive for CD138, CD45, CD45R, and CD20, and negative for CD10 and CD34); DLCL-like (A), negative for all markers tested (CD3, CD10, CD20, CD45, CD45R, CD138, and cytokeratin); DLCL-like (B) lymphoma CD45-positive but negative for all other markers; DLCL-like (C), CD138- and CD45-positive, and negative for all other markers. Osteosarcoma (A) osteoblastic by morphology, (B) more differentiated by morphology; MFH-like (A) angiomatoid type, (B) non-angiomatoid type

To address whether mΔpro can overcome the oncogenic effects of Δ122p53, a cohort of Δ122p53/mΔpro mice was monitored for 600 days. New cohorts of other Δ122p53 and mΔpro carrying genotypes were also monitored as controls. The Δ122p53/mΔpro cohort showed a unique survival curve with some animals developing tumors early, while others survived much longer (Figure 2a). Survival times ranged from 92 to 495 days with a median survival time of 275 days. The other cohorts of different genotypes showed similar survival kinetics to those previously published.^19, 28^ The survival of Δ122p53/mΔpro mice was significantly different to all other cohorts (*P*<0.001 for all comparisons).

Thus, although Δ122p53/mΔpro mice still develop tumors and have a shortened lifespan compared with p53^+/+^ mice, they are less tumor-prone and survive better than both Δ122p53/- and mΔpro/- mice. These data suggest that, and contrary to our initial hypothesis, mΔpro is capable of reducing the oncogenicity of Δ122p53 or Δ122p53 is able to enhance the tumor-suppressor properties of mΔpro.

### Δ122p53/mΔpro mice display a complex tumor spectrum

Next, histological and immunocytochemical analyses were carried out on the Δ122p53/mΔpro mice to determine their tumor spectrum. Results (Figure 2b) showed that the mice developed a range of tumors. Diffuse large B-cell lymphoma (DLCL), positive for the B-cell marker CD45R, was the most prevalent tumor type (27%) followed by sarcoma (21%). Ten percent of mice did not have a malignant tumor at necropsy, but developed hamartomas instead. Genotyping analysis showed that Δ122p53/mΔpro malignant tumors retained both the Δ122p53 and mΔpro alleles, whereas all six benign tumors retained the Δ122p53 allele, but lost the mΔpro allele, (data not shown). With regard to the DLCL and DLCL-like tumors, further immunophenotyping revealed six tumor sub-types (described in greater detail in the legend to Figure 2).

The complex tumor spectrum evident in the Δ122p53/mΔpro mice is very similar to the spectrum observed for Δ122p53/+ mice (Figure 2b) and homozygous Δ122p53 mice previously reported^19^ and completely unlike the tumor spectrum of mΔpro homozygous mice.^28^ Thus, as the presence of mΔpro does not alter the tumor spectrum of the mice, it seems likely that Δ122p53 is enhancing the ability of mΔpro to prevent tumor onset caused by Δ122p53 but not to alter its cancer-causing properties.

### The tumor spectrum of Δ122p53/mΔpro mice changes over time

**Figure 3:**
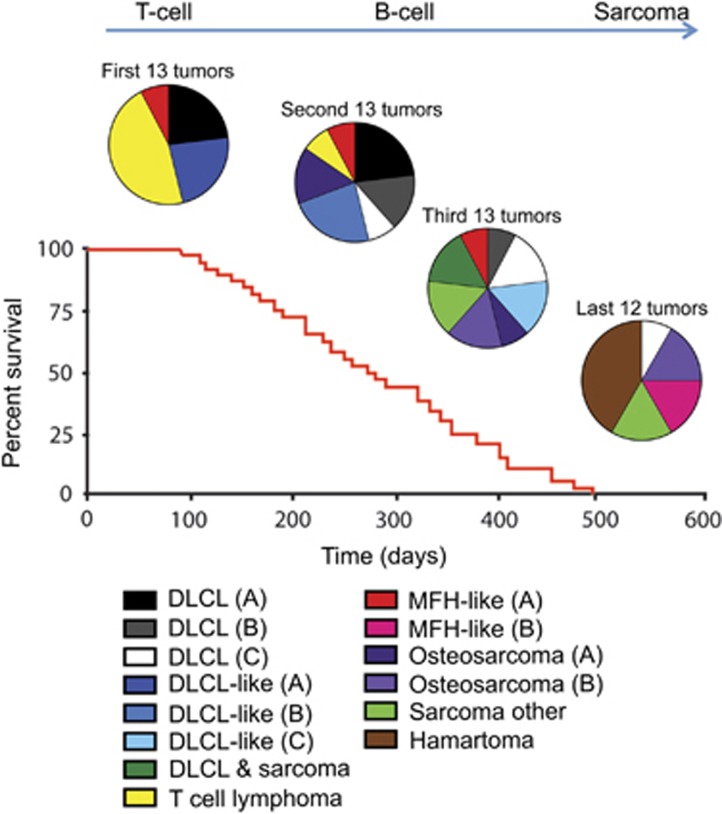
As Δ122p53/mΔpro mice age, different tumor types become predominant. The spontaneous tumor spectrum of the Δ122p53/mΔpro mice from Figure 2 was divided into four groups based on survival time: the first 13, the second 13, the third 13, and the last 12 mice to be killed because of tumor burden, to illustrate the predominance of different tumors types at different times. The classification: DLCL, DLCL-like, osteosarcoma and MFH-tumors were subdivided based on morphological or cell surface markers using immunohistochemistry as outlined in the legend to Figure 2

Unlike other genotypes, the survival times of Δ122p53/mΔpro mice are very broad ranging from 92 to 495 days. We therefore asked whether the tumor type varied with time. We found that different tumor types became more prevalent as the animals aged (Figure 3). DLCL or DLCL-like tumors and T-cell tumors were predominant in the early tumor onset group. T-cell tumors were the predominated tumor type in the first 15 mice to succumb to tumors, but no T-cell tumors were found in mice that survived past day 231. In the 21 mice that survived the longest, 33% developed sarcoma, 24% hamartoma, 19% DLCL, or DLCL-like tumors (all the more differentiated sub-types), and 14% developed malignant fibrous histocytoma.

In summary, Δ122p53/mΔpro mice that were killed early because of tumor burden had a similar tumor spectrum, aggressive B- and T-cell tumors, compared with Δ122p53/- and mΔpro/- mice. However, Δ122p53/mΔpro mice that survived longer had a different tumor spectrum with more differentiated lymphomas, sarcomas, and benign tumors. Therefore, it seems likely that Δ122p53 augments the ability of mΔpro to provide protection predominantly against early onset lymphoma formation.

### Cells from Δ122p53/mΔpro mice showed enhanced cell cycle arrest in response to DNA damage

**Figure 4:**
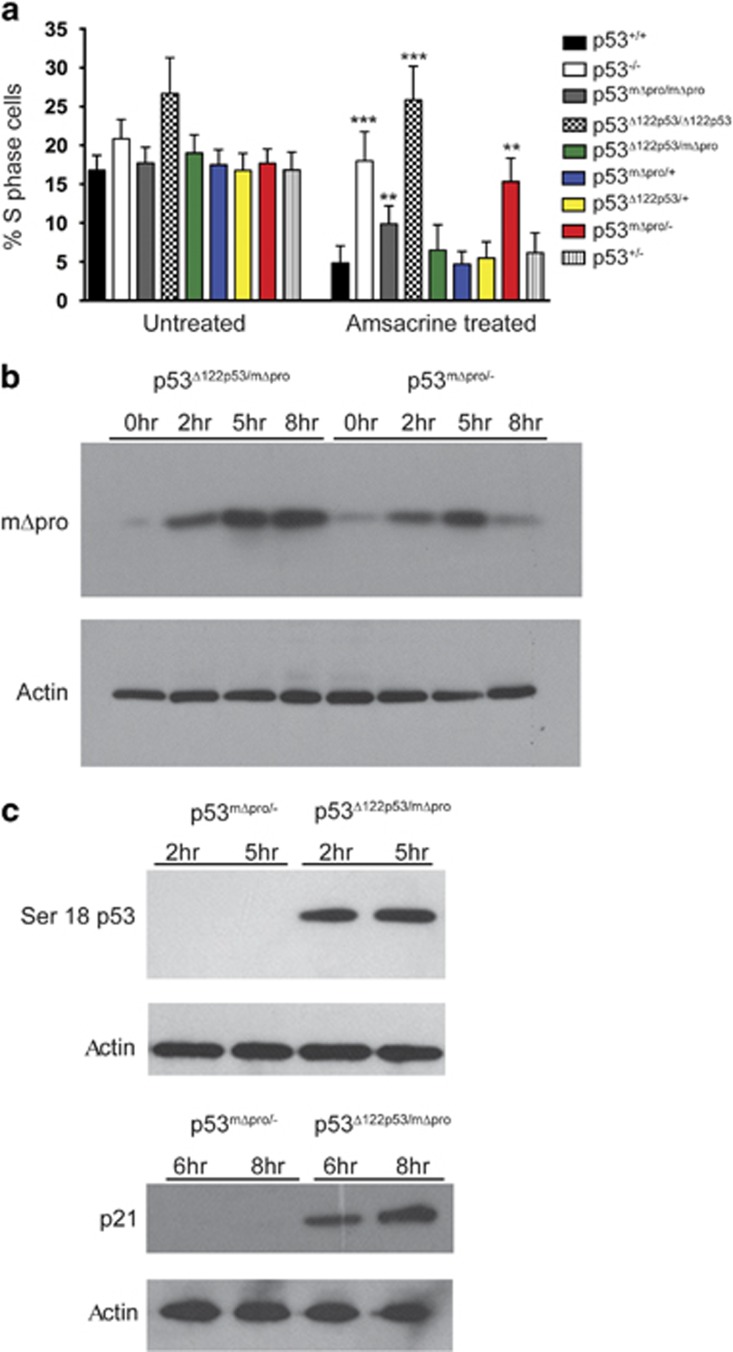
Δ122p53 stabilizes mΔpro and enhances its ability to induce a cell cycle arrest after DNA damage. (**a**) Bone marrow from Δ122p53/mΔpro mice induced a cell cycle arrest response following DNA damage. Bone marrow was isolated from 4 to 6-week-old mice of indicated genotypes, cultured and treated with 0.2 *μ*g/ml amsacrine. After 24 h, cells were pulse-labeled with BrdU, harvested, fixed, and stained with a fluorescent antibody to BrdU and the percentage of BrdU-positive cells was measured by flow cytometry. Bone marrow from mice with various combinations of the Δ122p53, mΔpro, wild-type (+), or p53 null (-) alleles were included for comparison. ****P<0.0001, ***P<0.001, **P<0.01, *P<0.05 in comparison with p53^+/+^ treated. Results are represented as the mean±S.D.; *n*=6 mice per genotype. (**b**) The presence of the Δ122p53 allele, stabilized mΔpro after DNA damage. Splenocytes from Δ122p53/mΔpro and mΔpro/- mice were cultured, exposed to 1 *μ*g/ml amsacrine and western blots carried out with an antibody to the N terminus of p53 to detect mΔpro. (**c**) The presence of the Δ122p53 allele led to increased Ser18 phosphorylated mΔpro (left) and increased p21^CIP1^ (right) in response to DNA damage. Splenocytes from Δ122p53/mΔpro and mΔpro/- mice were cultured, exposed to 1 *μ*g/ml amsacrine and western blots carried out with an antibody to phosphorylated Ser18 on p53 or p21^CIP1^. All experiments were carried out at least three times

To test whether the elevated tumor-suppressor functions of Δ122p53/mΔpro could be due to an enhanced ability to cause cell cycle arrest, bone marrow from Δ122p53/mΔpro mice and from mice of other genotypes were treated with amsacrine or left untreated. Amsacrine^30^ inhibits topoisomerase 2^31^ giving rise to double and single strand DNA breaks inducing a robust p53 response.^28, 29^ Untreated control cells from Δ122p53/mΔpro mice had a similar proportion of S-phase cells to those from homozygous Δ122p53 mice, but following amsacrine treatment, proliferation of Δ122p53/mΔpro cells was reduced to the same levels as seen in cells from p53^+/+^ mice (Figure 4a). Δ122p53/mΔpro also showed an improved ability to inhibit proliferation compared with mΔpro/- (*P*=0.0075, Figure 4a).

### Δ122p53 stabilizes mΔpro following DNA damage

Next, we determined whether the enhanced p53 response observed in Δ122p53/mΔpro cells could be explained by higher mΔpro. Levels of mΔpro were determined by western blotting from mΔpro/- and Δ122p53/mΔpro spleen lysates at 2, 5, and 8 h following amsacrine treatment using an N-terminal p53 antibody (which cannot detect Δ122p53). Results (Figure 4b) showed elevated levels of mΔpro in the Δ122p53/mΔpro lysates compared with mΔpro/- lysates after amsacrine treatment, which remained elevated after mΔpro alone had declined (8 h after treatment). In addition to total levels of mΔpro, we determined whether the activated form of mΔpro was elevated in the presence of Δ122p53 using western blotting with an antibody to phosphoserine 18 (p53ser18). Results (Figure 4c) show that phosphoserine mΔpro was detectable in the presence of Δ122p53 but not in its absence. Furthermore, on the same lysates, the cyclin-dependent kinase inhibitor (p21^CIP1^) was also detectable in the presence of Δ122p53 but not in its absence (Figure 4c). Similar results have been obtained in three separate experiments.

Taken together, the enhanced cell cycle arrest observed in the presence of Δ122p53 is likely due to Δ122p53 increasing mΔpro stability resulting in elevated p21^CIP1^.

### High levels of mouse Δ122p53 and human Δ133p53*α* stabilize FLp53 but this leads to an inhibition of p21^CIP1^

**Figure 5:**
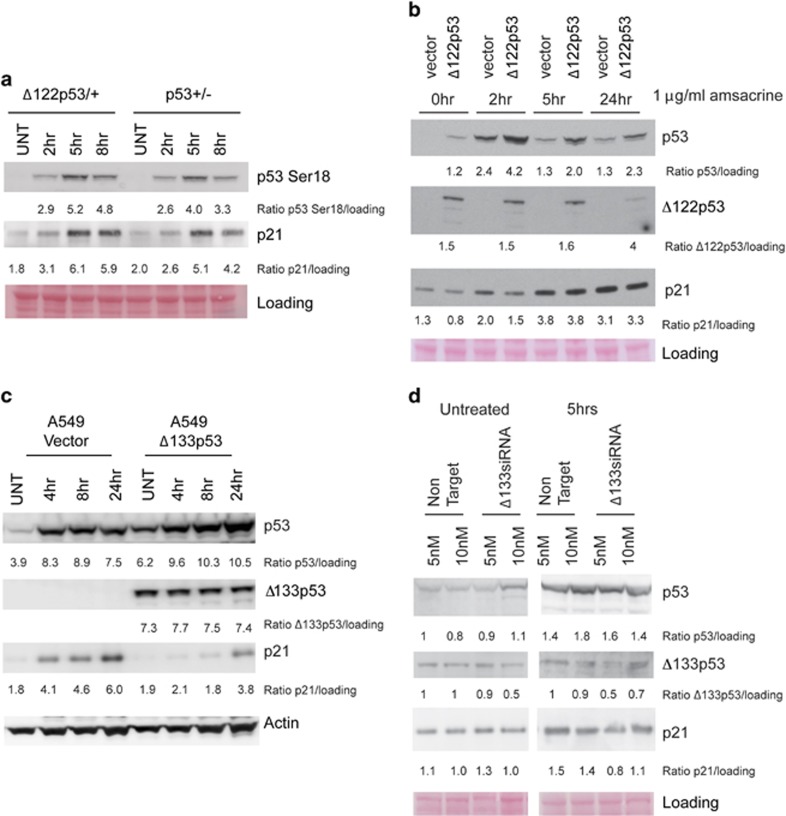
Δ122p53 and Δ133p53*α* stabilize FLp53 but inhibit FLp53 activity. (**a**) Splenocytes from Δ122p53/mΔpro and mΔpro/- mice were cultured, exposed to 0.2 *μ*g/ml amsacrine, and western blots carried out with an antibody to phosphorylated residue 18 of p53 (Serine 18) and an antibody to the p53 target gene, p21^CIP1^. Equal loading was determined by Ponceau S staining. (**b**) Mouse 3T3 cells were transduced with either an empty vector or a retroviral vector expressing Δ122p53. The transduced cells were then exposed to 1 *μ*g/ml of amsacrine and western blotting carried out for p53, Δ122p53 and p21^CIP1^. (**c**) A549 cells stably transduced with either an empty vector or Δ133p53*α* were exposed to 1 ug/ml amsacrine and harvested at the indicated time points and protein levels determined by western blotting. (**d**) A549 cells were transfected with either non-targeting siRNA or siRNA targeting Δ133p53 for 48 h, treated with 1 *μ*g/ml of amsacrine for 5 h, then protein levels determined by western blotting

As the data show that mΔpro is stabilized by Δ122p53, we were interested to know whether FLp53 is also stabilized. To this end, we isolated splenocytes from p53^+/−^ mice and heterozygous Δ122p53/+ mice. Results (Figure 5a) show that FLp53 is stabilized after amsacrine treatment to a greater level in Δ122p53/+ than p53^−/+^ cells. Quantitation suggests this is approximately 30–50% higher in the presence of Δ122p53, which, although small, was reproducible (*n*=3). There was also a similar and sustained increase in p21^CIP1^ protein levels post treatment in Δ122p53/+ cells, compared with p53^+/−^ cells.

To determine whether Δ122p53 can stabilize FLp53 in a different cellular context, we treated mouse 3T3 cells transduced with either an empty vector or a vector encoding Δ122p53, with amsacrine. The results in Figure 5b show that FLp53 is stabilized following amsacrine treatment reaching maximal levels at 2 h before subsiding, but was generally enhanced by approximately twofold in the presence of Δ122p53. However, in contrast to the splenocytes, there was a transient decrease in the levels of p21^CIP1^ in the Δ122p53-transduced cells suggesting that when overexpressed, Δ122p53 can inhibit FLp53 function.

To investigate whether human Δ133p53*α* also stabilizes FLp53, we used A549 lung cancer cells that had been transduced with a retrovirus expressing Δ133p53*α*.^19^ Results (Figure 5c) show that Δ133p53*α* stabilizes FLp53, however, like transduced Δ122p53, transduced Δ133p53*α* caused a decline in p21^CIP1^ levels, although this substantially recovered by 24 h after treatment. Given that the overexpression of Δ133p53 and Δ122p53 led to lower levels of p21^CIP1^, in contrast to the results observed with endogenous levels of Δ122p53, we hypothesized that the levels of Δ133p53/Δ122p53 were important. Some support for this is shown in Figure 5d in which the endogenous levels of Δ133p53*α* were reduced with siRNAs in untransduced A549. Figure 5d shows about a 50% decrease in Δ133p53 levels upon amsacrine treatment, which led to a 30–50% reduction in p21^CIP1^. These data suggest that endogenous levels of Δ133p53*α* are stimulatory of FLp53, rather than inhibitory as seen in cells transduced with Δ133p53*α* expression constructs. Thus, although this requires more detailed investigations, it appears that when Δ122p53 and Δ133p53*α* concentrations are low, they can cooperate with FLp53, but they are inhibitory at higher concentrations.

We also investigated whether the stabilization of FLp53 by Δ122p53 increased apoptosis after DNA damage in mouse tissues. Results from analysis of spleen and thymus tissues from Δ122p53/+, compared with heterozygous p53^+/−^ mice, show that the presence of the Δ122p53 allele has no impact on the ability of FLp53 to induce apoptosis (Supplementary Figure 1).

Collectively, the experiments suggest that Δ122p53 (and Δ133p53*α*) has two kinds of interaction with p53 (FLp53 or mutant)—the first is to interfere with normal p53 degradation and the second is to modulate p53 dependent transactivation.

**Figure 6:**
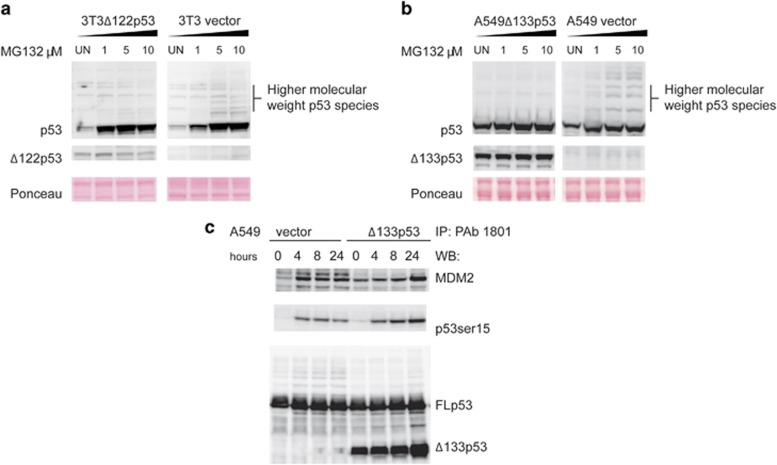
Δ122p53 and Δ133p53*α* inhibit proteasomal degradation of FLp53. (**a**) Mouse 3T3 cells transduced with either an empty vector or Δ122p53 were treated with the proteasomal inhibitor MG132 at the indicated concentrations for 4.5 h. Following MG132 treatment, cells were harvested and protein levels determined by immunoblotting. (**b**) Experiments were repeated using human A549 cells transduced to express Δ133p53*α*. (**c**) A549 cells transduced with Δ133p53*α* or a vector control were treated with 1 *μ*g/ml amsacrine for 0, 4, 8, and 24 h. Following amsacrine treatment, cells were harvested and subjected to immunoprecipitation with the p53 antibody pAb1801, followed by western blotting with a rabbit polyclonal p53 phospho-serine antibody to detect activated p53; the p53 antibody pAb240 to detect Δ133p53*α*; and SMP14 to detect bound MDM2

To investigate whether impaired MDM2 function could explain the stabilization of mΔpro and FLp53, the cells transduced with Δ122p53 or Δ133p53*α* were treated with increasing concentrations of the proteasome inhibitor MG132, lysates prepared, and western blotting for p53 carried out. Results for cells transduced with the vector show (Figure 6) that in the presence of MG132, p53 was stabilized as indicated by a series of high molecular mass protein species that increased with dose. Although a similar pattern was observed in cells expressing Δ122p53 (Figure 6a) or Δ133p53*α* (Figure 6b), the presence of the higher mass proteins was markedly reduced. These data suggest that proteasome-dependent degradation of p53 is inhibited by co-expression of Δ122p53 and Δ133p53*α*.

To test whether the expression of Δ133p53*α* inhibits the binding of MDM2, hence leading to stabilization of FLp53, the A549 cells transduced with Δ133p53*α* and the vector control were treated with amsacrine, harvested, and protein lysates prepared. Immunoprecipitation was then carried out using the p53 antibody, pAb 1801, which binds to the N-terminus of FLp53 (thus, it is unable to immunoreact with Δ133p53*α*), followed by western blotting with antibodies to MDM2, p53, and phosphorylated p53 and pAb 240 to detect Δ133p53*α*. Results show (Figure 6c) that Δ133p53*α* is bound to FLp53 as expected with more binding at later times after amsacrine treatment, and similar results were found for MDM2. Thus, although Δ133p53*α* can inhibit the degradation of FLp53, it does not do this by preventing MDM2 binding.

## Discussion

Using a mouse model of the Δ133p53*α* isoform (Δ122p53), we previously showed that the isoform had powerful tumorigenic and inflammatory functions, and in heterozygous mice, could partially inhibit the tumor-suppressor activities of wild-type p53.^19^ In this paper, we report that Δ122p53 enhanced the tumor-suppressor activities of the attenuated p53 mutant mΔpro. This was shown by the observation that Δ122p53/mΔpro mice survived much longer than mΔpro/- and Δ122p53/- mice and were largely protected from the aggressive early onset T-cell lymphomas typical of p53^−/−^ mice, and the least differentiated DLCL and DLCL-like tumors common in Δ122p53/- mice. Δ122p53/mΔpro mice that were killed early had a tumor spectrum similar to these mice but the longer term survivors developed multiple tumors, but predominantly the more differentiated DLCL and DLCL-like tumors, and non-lymphoid tumors including benign tumors, such as hamartomas. Why such diverse tumor types develop in Δ122p53/mΔpro mice is unclear. There might be an age component as p53^+/−^ mice live longer than p53^−/−^ mice, develop fewer lymphomas, and have a more complex tumor spectrum,^26^ although loss of the wild-type allele was common in p53^+/−^ tumors, unlike our Δ122p53/mΔpro tumors. Thus, some level of p53 activity reduces the chance of developing early onset lymphoma, but is insufficient to prevent other tumor types. p53 forms tetramers that can include other p53 isoforms, p63, or p73 family members.^32^ It is therefore possible that different hetero- and homo-dimeric complexes of Δ122p53 and mΔpro exist, and in different ratios, can either protect against tumor development or not, and that this is a largely stochastic process.

The finding that Δ122p53 increased the lifespan of Δ122p53/mΔpro mice, and protected these mice from tumor development was unexpected. All functions attributed to Δ133p53*α* to date suggest a pro-tumorigenic phenotype—it is anti-apoptotic,^11, 33^ pro-proliferative,^16, 34, 35^ and pro-angiogenic.^17^ Our earlier study showed Δ122p53 to be pro-proliferative and pro-inflammatory, consistent with the pro-tumorigenic phenotype of Δ133p53*α*.^19^ To explain this paradox, we carried out *in vitro* studies with cells derived from Δ122p53/mΔpro mice. We found that Δ122p53 improved the response of mΔpro to DNA damage by increasing the ability of mΔpro to induce cell cycle arrest, probably by stabilizing mΔpro protein. Thus, arrest and repair are most likely to be the tumor-suppressor mechanisms used by mΔpro. However, as serum levels of IL-6 are elevated in Δ122p53/mΔpro mice compared with other genotypes (Figure 1c), and IL-6 is a marker of the so-called senescence associated secretory phenotype,^36^ we looked for evidence of senescent cells in the tumor sections from the mice by immunostaining for the senescence marker p16^INK4A^.^37^ None was found (data not shown). Thus, although we cannot exclude senescence as being a tumor-suppressor mechanism, it seems unlikely.

In addition to mΔpro being stabilized, we also found this to be the case for FLp53, although to a lesser extent. We therefore investigated the mechanism of stabilization. Our data suggest that it is likely due to the ability of Δ122p53 and Δ133p53*α* to inhibit proteasomal degradation of FLp53. However, this appears to be independent of MDM2 binding as Δ133p53*α* does not inhibit MDM2 from interacting with FLp53. Despite being stabilized, FLp53 tumor-suppressor functions are not enhanced by Δ122p53 or Δ133p53*α*, presumably because they are already maximal, or the degree of stabilization is insufficient to elicit a biological response.

Enhanced FLp53 function by an N-terminal-deleted p53 has also been reported for the Δ40p53 isoform.^38^ In Saos-2 cells transfected with different amounts of FLp53 and Δ40p53, p53 transcriptional activity was slightly increased when Δ40p53 was at low concentrations compared with FLp53 alone, but FLp53 activity was strongly inhibited when higher concentrations of Δ40p53 were co-expressed. These data are very similar to the data we show here for Δ122p53. Thus, the ratio of the isoforms to FLp53 is important. Also consistent with the current study, the presence of Δ40p53 led to increased concentrations of phosphorylated serine 15 and prevention of FLp53 degradation by MDM2. In this study, the cellular context was found to be important as similar findings were not obtained using H1299 cells.

In summary, the combination of the Δ122p53 and mΔpro alleles rescued many animals from aggressive early onset tumors—an unexpected result—given the weak tumor-suppressor activity of mΔpro and the oncogenic properties of Δ122p53. One explanation for the improved survival could be a role for Δ122p53 in enhancing and prolonging the mΔpro response to DNA damage by inhibiting p53 degradation leading to enhanced ability to induce cell cycle arrest. Given the similarities between Δ122p53 and Δ133p53*α* as shown here and in our previous work,^19^ we propose that at certain ratios of Δ133p53*α* to FLp53, Δ133p53*α* can elevate the response of p53 to DNA damage and thus in some circumstances, increase p53 tumor-suppressor activity.

## Materials and Methods

### Mice

The Δ122p53 mice and mΔpro were constructed as previously described.^19, 28, 29^ All mice were on the C57/BL6 background. Δ122p53/mΔpro mice were created by crossing Δ122p53 and mΔpro mice.

### Cell lines

Mouse 3T3 Δ122p53 and 3T3 vector cells,^19^ and human A549 Δ133p53*α* and A549 cells were described previously.^19^

### MG132 treatment

Prior to treatment, cells were seeded in six-well plates. The proteasome inhibitor MG132 (Sigma Aldrich, St. Louis, MO, USA) was diluted to concentrations from 0.5 to 10 *μ*M. The cells were incubated with media containing MG132 for 4.5 h and then harvested to produce lysates.

### Western blotting

Splenocytes were isolated from 4 to 6-week-old animals and treated with amsacrine at indicated concentrations or the vehicle control, and incubated in complete RPMI media as indicated. Cell lines were seeded and cultured, treated as indicated and incubated for different times as indicated in the legends to the figures. Protein lysates were prepared in the presence of protease inhibitors, with 20–40 *μ*g of protein separated on NuPAGE 4–12% Bis-Tris Gels (Life Technologies, Carlsbad, CA, USA). Blots were probed with primary antibodies against the *N*-terminus of p53 (1C12, Cell Signaling Technology, Boston, MA, USA), Phospho-p53 (Ser15) (9284, Cell Signaling), FL393 to detect Δ133p53*α*, p21 (C-19, Santa Cruz Biotechnology, Santa Cruz, CA, USA) and β-actin (AC-15, Abcam, Cambridge, UK) according to the manufacturers' instructions. Alkaline phosphatase-conjugated antibodies were detected using the Western Breeze Immunodetection kit (Life Technologies). Results were repeated three times per genotype.

### Cell cycle arrest/proliferation analysi*s*

The *in vitro* and *in vivo* assays were carried out as previously described and used BrdU-pulsed cells^28^ and tissues.^19^ Four mice per genotype were assessed.

### Cytokine analyses by ELISA

Serum from 5 to 6-week-old animals was added to the Mouse IL-6 Quantikine ELISA kit (R&D Systems, Minneapolis, NE, USA) to measure IL-6, or the mouse IFN gamma ELISA kit (Pierce, Rockford, IL, USA) to measure *γ̃*IFN according to the manufacturers' instructions.

### *In vivo* apoptosis assay

Male mice (12–13 weeks old) of the specified genotypes were treated with 5 Gy whole-body irradiation and killed at the indicated time points after irradiation by cervical dislocation. Samples were processed for cytometric cell cycle analysis according to our established method^39^ following the protocol published in the study by Heinlein and Speidel.^40^ Analysis of propidium iodide-stained cells was performed on a Beckman Coulter FC-500 instrument (Beckman Coulter, Pasadena, CA, USA). The percentage of subG1 fragments was determined using CXP software (Beckman Coulter). Error bars are standard deviation.

### Survival and pathological analyses

Mice were aged for 600 days and killed when visible tumor burden was apparent or at the end of the study period (day 601).^28^ Immunohistochemisty analyses were performed on tumor sections to confirm histopathological examinations and further sub-typing of tumors was carried out using the following primary antibodies: CD3 (ab5690, Abcam), CD10 (ab951, Abcam), CD34 (ab8158, Abcam), CD45R (clone RA3-6B2, BD Biosciences, Franklin Lakes, NJ, USA), CD45 (clone 30-F11, BD Biosciences), and CD138 (ab34164, Abcam).

### Tumor DNA analysis

The Δ122p53 and mΔpro alleles were amplified using PCR from DNA extracted from paraffin-embedded tumors as previously described.^19, 28^

### Statistical analyses

Results are expressed as the mean±S.D. Unless otherwise stated, results are from at least three independent experiments with at least two mice per genotype in each experiment. Statistical differences between two groups were evaluated using the Student's *t*-test, with *P*<0.05 taken as a significant difference.

## Abbreviations

BrdU: bromodeoxyuridine
CDKN1*α*: cyclin-dependent kinase inhibitor 1 alpha
DLCL: diffuse large B-cell lymphoma
ELISA: enzyme-linked immunosorbent assay
FL: full-length
*γ*-IFN: gamma interferon
IL-6: interleukin-6
MDM2: mouse double minute 2 homolog
MFH: malignant fibrous histocytoma
p53: tumor protein 53
PRD: proline-rich domain
