# Supplementary figures and images for: Δ122p53, a mouse model of Δ133p53α, enhances the tumor-suppressor activities of an attenuated p53 mutant
Source: Cell Death Dis. 2015 Jun 11;6(6):e1783–. doi: 10.1038/cddis.2015.149 (PMC4669831; doi:10.1038/cddis.2015.149)

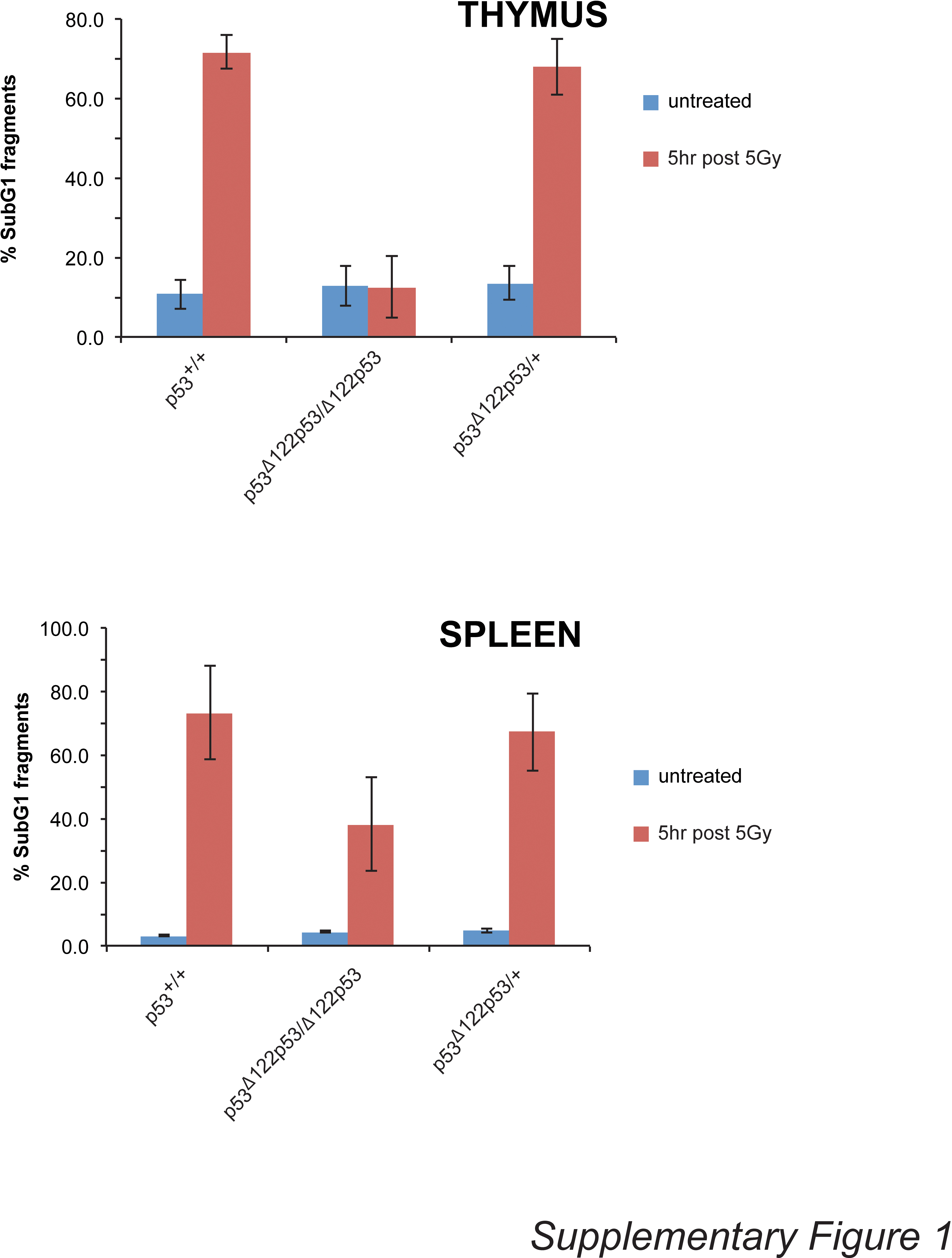

Supplement: Supplementary Figure 1 [file cddis2015149x1.tif]
