# Supplementary material for: Δ122p53, a mouse model of Δ133p53α, enhances the tumor-suppressor activities of an attenuated p53 mutant
Source: Cell Death Dis. 2015 Jun 11;6(6):e1783–. doi: 10.1038/cddis.2015.149 (PMC4669831; doi:10.1038/cddis.2015.149)
Supplement: Supplementary Figure Legend [file cddis2015149x2.doc]

**Supplementary Figure 1. Δ122p53 does not block the ability of FLp53 to induce apoptosis *in vivo*.** Mice of the indicated genotypes received 5Gy whole-body irradiation or mock treatment and their spleens and thymi were analyzed for apoptotic subG1 fragments. Bars represent the average from 3 mice per treatment and genotype: error bars represent the standard deviation of the mean.
